# The Effect of Wearing a Lower Body Compression Garment on Anaerobic Exercise Performance in Division I NCAA Basketball Players

**DOI:** 10.3390/sports7060144

**Published:** 2019-06-13

**Authors:** Christopher Ballmann, Hunter Hotchkiss, Mallory Marshall, Rebecca Rogers

**Affiliations:** Department of Kinesiology, Samford University, Birmingham, AL 35229, USA; hhotchkiss11@gmail.com (H.H.); mmarshal@samford.edu (M.M.); rrogers1@samford.edu (R.R.)

**Keywords:** wingate, compression, anaerobic capacity, anaerobic power, basketball

## Abstract

Lower body compression (LBC) has been shown as an effective recovery tool from basketball but it is unknown how it affects performance. The purpose of this study was to examine the effects of wearing a LBC garment on anaerobic exercise performance in collegiate basketball players. Healthy Division I collegiate basketball players (n = 12) were recruited for this study. In a crossover, counterbalanced study design, subjects volunteered to participate in two separate visits each with a different condition: wearing a LBC garment or non-compressive control (CON) garment. During each visit, subjects completed 2 × 30 second Wingate Anaerobic Tests (WAnTs) separated by a 5-min active recovery period. Each visit was separated by a 72 h washout period. Results revealed that over the 2 × 30 second WAnTs, mean power output (*p* = 0.028; d= 0.35), anaerobic capacity (*p* = 0.018; d = 0.45), and total work (*p* = 0.027; d = 0.36) were higher when wearing the LBC versus CON garment. However, peak power output (*p* = 0.319; d = 0.09), anaerobic power (*p* = 0.263; d = 0.23), and fatigue index (*p* = 0.749; d = 0.05) were not statistically different. Rating of perceived exertion (RPE) was significantly lower (*p* = 0.032; d = 0.72) with LBC compared to CON. Results indicate that LBC may increase anaerobic exercise performance in collegiate basketball players.

## 1. Introduction

Many competitive sports involve high intensity anaerobic bouts of exercise that are usually repetitive in nature. Better recovery in between these bouts of exercise may ultimately lead to greater performance. Various different methods to enhance recovery and performance during anaerobic exercise have been studied, including passive and active recovery [1,2], ergogenic aids [3], nutritional supplementation [4], and cryotherapy [5]. Recently, the use of compression garments, specifically lower body compression (LBC), has gained popularity for use during and after exercise in efforts to aid in recovery [6]. However, much of the evidence for LBC efficacy is conflicting and evidence of LBC use to aid in anaerobic performance in particular is lacking.

Much of the evidence supporting the use of LBC focuses on post-exercise recovery and combating exercise-induced muscle soreness [7,8,9,10]. For example, Jakeman et al. reported that LBC following strenuous exercise preserved knee extensor strength and reduced perceived muscle soreness [8]. LBC has also been used as a recovery tool to aid in performance with subsequent exercise bouts. In one study, de Glanville et al. showed that wearing a LBC garment for 24 h following a 40 km cycling time trial improved subsequent 40 km trial performance and resulted in higher average power output when compared to a placebo garment [7]. However, other studies have shown trivial improvements in recovery of muscle performance measures [9,11]. Mechanisms for changes in subsequent exercise performance are not fully understood, but are likely due to hemodynamic changes in which LBC may have beneficial effects on venous flow [12]. Furthermore, there is evidence to suggest that LBC may enhance tissue oxygenation during recovery [13]. While using LBC as a recovery tool has been well supported, use of LBC during exercise to enhance performance is less clear.

Wearing LBC during exercise to enhance performance has largely been studied in endurance-based aerobic activities and has revealed mixed results [14,15,16,17]. Kemmler et al. reported lower leg compression stockings significantly improved running performance at multiple metabolic thresholds [14]. Furthermore, Driller et al. reported LBC significantly enhanced power output during cycling exercise, albeit the effect was small [16]. However, these findings have been refuted by multiple investigations, leaving the actual contribution of LBC to enhance exercise performance equivocal [15,17]. Interestingly, however, there are a number of studies suggesting possible physiological benefits of wearing LBC during exercise without observed changes in performance. For example, Menetrier et al. showed LBC improved tissue oxygen saturation during exercise but did not observe a change in running performance [13]. Dascombe et al. also reported LBC increased lower body regional blood flow and oxygen unloading, despite no changes in running performance [17]. Taken together with previous evidence of LBC during recovery, the physiological changes may influence recovery from exercise to a greater extent than performance. However, there is comparatively less research investigating LBC and anaerobic exercise performance and the previously described physiological changes may affect anaerobic activities differently, which warrants further study.

The effects of LBC on anaerobic performance has been comparatively less studied. Kraemer et al. reported increases in vertical jump height with LBC in female volleyball players [18]. Faulkner et al. reported lower effort perception with LBC during a 400 m sprint but observed no changes in performance, and Hamlin et al. reported improved recovery and fatigue during repeated 40 m sprints in rugby players [19,20]. Given the conflicts between these results, further study of LBC on anaerobic exercise performance is warranted. Basketball is a sport that is largely anaerobic in nature, with elements such as rapid accelerations, sprints, and explosive jumping [4,21,22]. Thus, increasing performance of these elements may lead to better game play performance. LBC has been shown to be an effective recovery tool from basketball game-play in competitive male players [4]. However, to our knowledge performance during anaerobic exercise bouts while wearing LBC in competitive basketball players has yet to be studied. Thus, the purpose of this study was to examine the effects of wearing a LBC garment on anaerobic exercise performance in Division I NCAA basketball players. We hypothesized that LBC would aid in increasing and maintaining mean and peak anaerobic performance outcomes across repeated Wingate anaerobic tests (WAnT).

## 2. Materials and Methods

### 2.1. Study Design

Using a counterbalanced, crossover design, the following investigation studied the effects of LBC on anaerobic performance in collegiate basketball players. Male Division 1 NCAA basketball players volunteered to participate and completed 2 separate visits each with a different condition: wearing a LBC or non-compressive control (CON) garment. During each visit, subjects completed 2 × 30 second repeated Wingate anaerobic tests (WAnTs). Each visit was separated by a 72 h washout period. Conditions were compared according to anaerobic performance variables, including mean power, peak power, anaerobic capacity, anaerobic power, total work, and fatigue index. Test-to-test results and average performance across the two repeated WAnTs were analyzed.

### 2.2. Subjects

Twelve collegiate male basketball players (age (yrs) = 20.3 ± 1.37, height (cm) = 194.5 ± 8.9, body mass (kgs) = 88.8 ± 11.4) were recruited for this investigation. In order to participate, subjects had to be on a current roster of an NCAA Division 1 basketball team. All subjects completed a physical activity readiness questionnaire (PAR-Q) to determine suitability for exercise. Subjects were excluded if they had a lower body injury in past six months. Subjects were asked to refrain from strenuous activity, alcohol, caffeine, and nicotine 24 h prior to testing. All data collection was recorded in the collegiate basketball off-season. All subjects gave their informed consent for inclusion before they participated in the study. The study was conducted in accordance with the Declaration of Helsinki, and the protocol was approved by the Samford University Institutional Review Board (IRB).

### 2.3. Procedures

Upon arrival, subject height and body mass were documented. Subjects then donned either a personally fitted LBC garment or CON garment for WAnT testing. Fitting of the compression garment was determined using height and body mass according to the manufacturer’s recommendations. The LBC garment (Nike, Beaverton, OR, USA) covered from the waist to the ankles and was composed of 80% nylon and 20% spandex. The gradient of compression went from approximately 15–20 mmHg at the ankle to 6–10 mmHg at the thigh (Nike, Beaverton, OR, USA). The CON garment was shorts, which provided no compression [16,19]. Seat height was adjusted for each subject to where the knee had approximately 5 degrees of flexion, while the crank of the cycle was at the bottom [23]. Seat height was recorded and used for all subsequent trials. Subjects completed a light warm-up on an electronically braked Veltoron cycle ergometer (Racermate Inc., Seattle, WA, USA) by pedaling at a comfortable pace against unloaded resistance for 5 min. Resistance for the WAnTs was calculated using 7.5% of the subject’s body mass. Subjects then achieved maximal pedaling rate through a 10-second acceleration phase before the resistance was applied [3]. Following the acceleration phase, the weight was immediately applied and subjects were verbally encouraged to pedal as hard and fast as possible for a total of 30 seconds. This was repeated one successive time with 5 min of rest in between each WAnT. Rating of perceived exertion (RPE; scale 1–10) was taken immediately after the completion of the 2nd WAnT.

### 2.4. Statistical Analysis

All data were analyzed using SPSS 25 (IBM, Armonk, NY, USA). A 2 × 2 (Condition × Test) repeated measures ANOVA was used to analyze test-to-test comparisons with a Tukey post hoc analyses if warranted. Average performance over the two WAnTs was analyzed using a paired sample t-test. Effect sizes for average performance data were calculated using Cohen’s d effect size (d) calculator for t-test and interpretation [24]. Data are presented as mean ± standard deviation (SD). Significance was set at *p* ≤ 0.05 a priori.

## 3. Results

**Figure 1:**
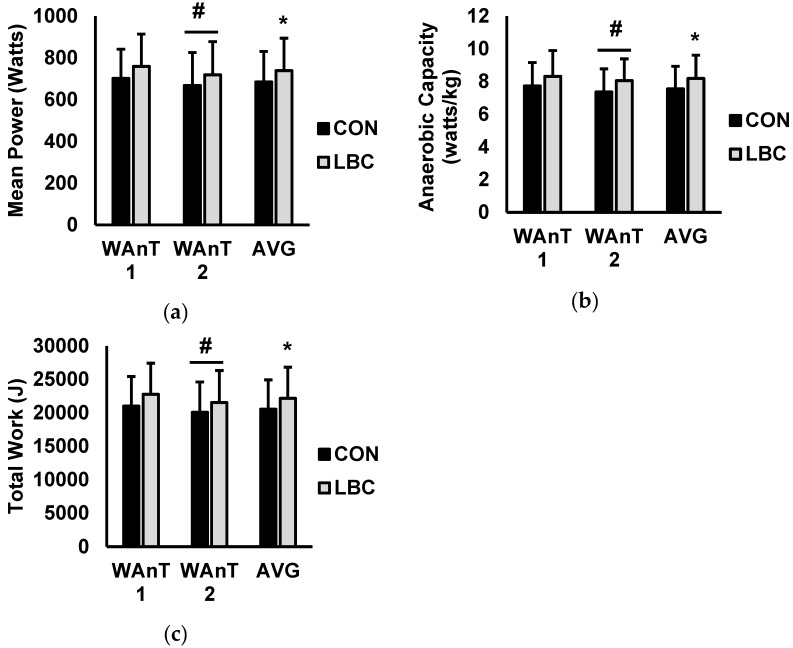
Data are presented as mean ± SD. Control (black) versus Lower body compression (LBC) (grey). (**a**) Mean power output, (**b**) anaerobic capacity, and (**c**) total work over Wingate anaerobic test (WAnT) 1, WAnT2, and averaged (AVG) over both WAnT 1 and WAnT2. Note: # denotes significantly different from WAnT 1 (*p* < 0.05); * denotes significantly different from control (*p* < 0.05).

**Figure 2:**
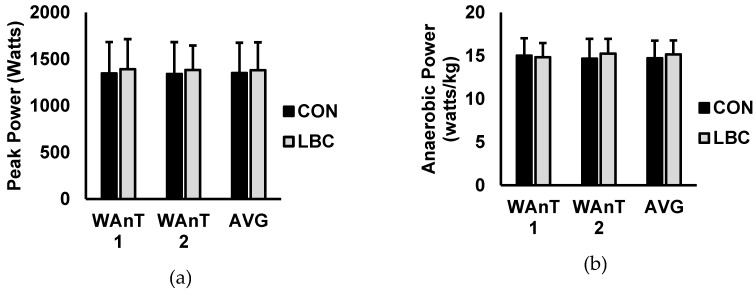

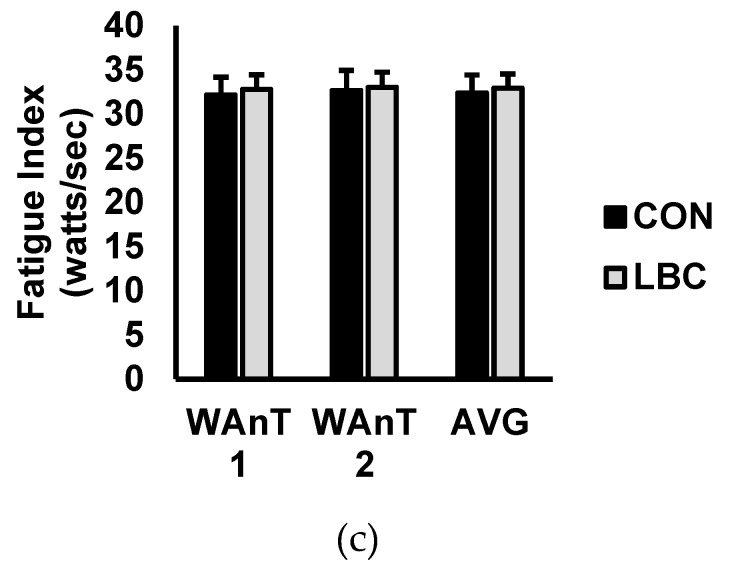
Data are presented as mean ± SD. Control (black) versus Lower body compression (LBC) (grey). (**a**) Peak power output, (**b**) anaerobic power, and (**c**) fatigue index over Wingate anaerobic test (WAnT) 1, WAnT2, and averaged (AVG) over both WAnT 1 and WAnT2.

**Figure 3:**
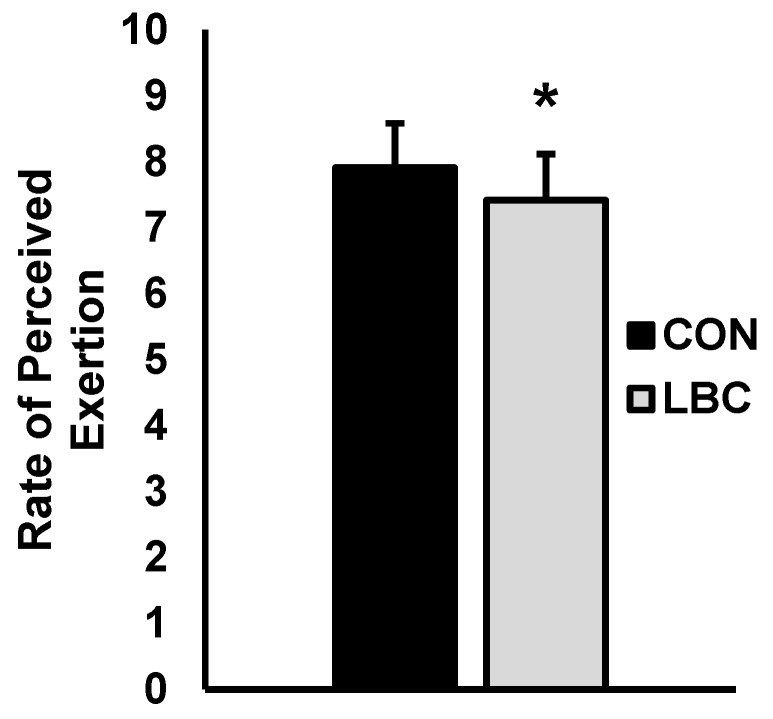
Data are presented as mean ± SD. Control (black) versus Lower body compression (LBC) (grey). Rating of perceived exertion (RPE) across repeated WAnTs. **Note:** * denotes significantly different from control (*p* < 0.05).

Test-to-test and average performance over the two WAnTs are presented in Figure 1 and Figure 2. For mean power (Figure 1A), there was a main effect for test (*p* = 0.001) but no effects for condition (*p* = 0.388) or interaction for condition and test (0.758). Average mean power was significantly higher while wearing LBC versus CON (CON = 684.5 ± 146.3 watts, LBC = 738.8 ± 155.3 watts; *p* = 0.028; d = 0.35). For anaerobic capacity (Figure 1B), there was a main effect for test (*p* = 0.028) but no effects for condition (*p* = 0.277) or interaction for condition and test (*p* = 0.686). Average anaerobic capacity was significantly higher while wearing LBC versus CON (CON = 7.5 ± 1.3 watts/kg, LBC = 8.1 ± 1.4 watts/kg; *p* = 0.18; d = 0.45). For total work (Figure 1C) there was a main effect for test (*p* = 0.003), with no effects for condition (*p* = 0.639) or interaction for condition and test (*p* = 0.387). Furthermore, average total work completed was higher during the LBC trial versus CON (CON = 20,533.3 ± 4392.2 joules, LBC = 22,165.4 ± 4661.3 joules; *p* = 0.027; d = 0.36). Peak power (watts; Figure 2A) showed no effects for test (*p* = 0.738), no effects for condition (*p* = 0.935), and no interaction for condition and test (*p* = 0.738). Average peak power was also unaffected by LBC when compared to CON (CON = 1350.2 ± 325.9 watts, LBC = 1381.0 ± 299.0 watts; *p* = 0.319; d = 0.09). For anaerobic power (Figure 2B), there were no effects for test (*p* = 0.876) or condition (*p* = 0.797) and no interaction between condition and test (*p* = 0.111). Average anaerobic power was not significantly altered by LBC (CON = 14.7 ± 2.0 watts/kg, LBC = 15.1 ± 1.6 watts/kg; *p* = 0.263; d = 0.23). Fatigue index (Figure 2C) showed no main effects for test (*p* = 0.747) or condition (*p* = 0.899) and no interaction for condition and test (*p* = 0.908). Average fatigue index was not significantly different between LBC and CON trials (CON = 32.4 ± 10.7 watts/sec, LBC = 32.9 ± 8.5 watts/sec; *p* = 0.749; d = 0.05). Rating of perceived exertion (RPE; Figure 3) was significantly lower for LBC versus CON (CON = 7.9 ± 0.6, 7.4 ± 0.7; *p* = 0.032; d = 0.72).

## 4. Discussion

LBC has been reported to improve recovery from exercise and increase endurance performance. The influence LBC has on anaerobic exercise performance has been comparatively less studied and LBC efficacy on anaerobic performance improvements is still debated. Basketball is a sport that is largely anaerobic in nature and LBC has been shown to improve recovery from game-play [4]. However, it is currently unknown whether LBC can improve anaerobic exercise performance in basketball players. Thus, the current study investigated wearing a LBC garment on WAnT performance in Division I NCAA basketball players. Findings from the current study suggest that LBC enhances mean power output, anaerobic capacity, and total work during across successive WAnTs. Rating of perceived exertion (RPE) was also lower while wearing LBC. However, peak power, anaerobic power, and fatigue index were largely unaffected. While the physiological mechanisms responsible for increases in anaerobic capability were not elucidated, these data suggest that LBC could possibly affect game-play through increased anaerobic performance.

RPE during strenuous exercise may affect the ability of an athlete to perform a greater volume of work or at a higher intensity during training or competition. Important to the following study, improvements in power output occurred with concurrent decreases in RPE while wearing LBC garments. Supporting our findings, Faulkner et al. reported LBC decreased RPE during 400 m sprint times [19]. Of particular note, the order of magnitude in which RPE changed was very similar between their study (~5% decrease) and the current study (~6% decrease). However, Faulker et al. did not observe improvements in 400 m sprint time [19]. Differences between findings may be due to differences in the mode of exercise testing in that they tested sprint time during running versus cycling. Future study is needed to compare effectiveness of LBC with different modes of exercise. Kraemer et al. reported LBC improved vertical jump in competitive collegiate volleyball players [18]. Our findings of increased power output over the two WAnTs are consistent with their findings of increased power production over 10 jumps [18]. Although the two populations of athletes in each study were different, both support the use of LBC in well-trained collegiate athletes to enhance power output during anaerobic activities. Whether the type of athlete or training experience affects the ergogenic benefits of LBC is currently unknown and further investigation is warranted for different sports and levels of training.

The physiological factors for our observed changes in power output and RPE remain unknown and cannot be answered from our data set alone. However, previous evidence using LBC may provide rationale for changes in anaerobic performance. Menetrier et al. reported that LBC can increase tissue oxygen saturation during exercise [13]. Given that oxygen delivery may limit aerobic adenosine triphosphate (ATP) production and aerobic ATP production influences phosphocreatine (ATP-PC) resynthesis [25], LBC compression may enhance ATP-PC resynthesis and energy production during anaerobic exercise leading to improvements in power output. Our results do not fully support this in that although subjects were able to maintain a higher average power output over both exercise bouts, fatigue index was similar, and analysis of test-to-test revealed that subjects tended to have decreases in performance on the second WAnT regardless of condition. Kraemer et al. also reported no changes in peak power during jumping but improved average performance over multiple jumps, suggesting improved recovery [18]. However, a recent study investigating LBC during sprint performance revealed no change in muscle oxygenation but saw increases in peak power [26]. Thus, contribution of muscle oxygenation and LBC to power output during anaerobic exercise is not fully clear and merits further study. Another mechanism by which LBC has been reported to impart ergogenic benefits is through improvements in muscle blood flow, thus increasing lactate clearance, which may in turn affect fatigue [26,27,28]. Most of this evidence supporting LBC and lactate removal has been reported during aerobic or endurance-based activities [27,29]. Comparatively less is known of how LBC influences lactate removal during anaerobic activities. However, Broatch et al. [26] recently reported LBC increased muscle blood flow and exercise performance but had no influence on blood lactate during repeated short sprints. Of particular note, these findings were reported using a similar mode of exercise and active population; albeit not well-trained athletes. Further research on how LBC impacts blood flow and lactate clearance in well-trained athletes during anaerobic exercise is needed and may hold important implications in describing how LBC impacts performance and recovery.

Although LBC positively influenced anaerobic exercise performance in the current study, how the results translate to actual game-play is unknown. However, previous evidence has suggested that WAnT performance may predict performance in basketball specific skills. Hoffman et al. showed that mean power output during WAnTs had a moderate positive correlation with line drill (also known as “suicides”) performance [30]. Furthermore, counter movement jump height also had a moderate positive correlation with WAnT performance [30]. Thus, findings of improved power output with LBC in the current study may hold important implications of improving players abilities to jump and run from goal-to-goal during a game. Our findings of decreases in RPE with LBC may also have important effects on game-play performance. Lyons et al. reported that higher levels of fatigue led to decreases in basketball-passing scores in both novice and expert players [31]. Furthermore, Leite et al. showed that basketball game formats (i.e., 3 × 3 vs. 5 × 5) where players reported lower RPE scores improved offensive efficacy [32]. Similar findings have been found in other sports as well. Royal et al. showed that as RPE increased during water polo skill drills, skill proficiency and technique were negatively impacted [33]. Decreases in RPE with LBC could, therefore, positively impact basketball specific skills and game-play. Future research should focus on how LBC may impact game performance and sports specific drill performance.

While the present study presents novel information regarding LBC and anaerobic exercise performance in competitive basketball players, there were several limitations to the study. First, the current study only measured performance measures and did not measure physiological variables that may have led to improvements in performance, so the mechanism by which anaerobic performance is enhanced is unclear. In addition, the current study design did not allow for blinding of treatments. Although multiple investigations have used the same approach [16,19], the control garment may not have replicated the LBC compression garment adequately. Thus, we cannot rule out a possible psychological component to the observed improvements. In conclusion, the current investigation indicates that LBC increases mean power output, anaerobic capacity, total work, and decreases perceived exertion in collegiate basketball players. Future studies should focus on identifying physiological mechanisms responsible for increased anaerobic performance and study how LBC affects basketball game-play.
